# Association between time in range 70-180 mg/dl in early stage and severity with in patients acute pancreatitis

**DOI:** 10.1186/s12902-023-01414-2

**Published:** 2023-07-26

**Authors:** Chuchen Meng, Jie Zhang, Ying Wang, Xinhua Ye, Shaohua Zhuang

**Affiliations:** 1 Department of Endocrinology, The Affiliated Changzhou No. 2 People’s Hospital of Nanjing Medical University, Changzhou, Jiangsu China; 2 Department of Gastroenterology, The Affiliated Changzhou No. 2 People’s Hospital of Nanjing Medical University, 29 Xinglong Road Changzhou, Jiangsu, 213000 China

**Keywords:** Acute pancreatitis, Blood glucose time in range, Severity, Diabetes, Glycosylated hemoglobin

## Abstract

**Background:**

It is not well understood whether glucose control in the early stage of acute pancreatitis(AP) is related to outcome. This study aimed to investigate the association between blood glucose time in range (TIR) of 70–180 mg/dL in the first 72 h(h) on admission and the progression of AP.

**Methods:**

Individuals admitted with AP to the Gastroenterology Department of the Affiliated Changzhou No.2 People’s Hospital of Nanjing Medical University between January 2017 and December 2021 were included and retrospectively evaluated. The percentage of TIR between 70 and 180 mg/dL in the first 72 h was calculated. According to the progress of AP at discharge, patients were divided into mild pancreatitis(MAP), and moderately severe acute pancreatitis (MSAP), or severe acute pancreatitis (SAP) groups. We examined the association between TIR or TIR ≥ 70% and AP severity using logistic regression models stratified by a glycosylated hemoglobin (HbA1c) level of 6.5%. Receiver operating characteristic (ROC) curves were generated to assess the ability of the TIR to predict MSAP or SAP.

**Results:**

A total of 298 individuals were included, of whom 35 developed MSAP or SAP. Logistic regression analyses indicated that TIR was independently associated with the incidence of more serious AP (odds ratio [OR] = 0.962, 95% CI = 0.941–0.983, *p* = 0.001). This association remained significant in individuals with HbA1c levels ≤ 6.5% (OR = 0.928, 95% CI = 0.888–0.969, *p* = 0.001). A TIR ≥ 70% was independently associated with reduced severity only in people with well-antecedent controls (OR = 0.238; 95% CI = 0.071–0.802; *p* = 0.020). TIR was not powerful enough to predict the severity of AP in both patients with poor antecedent glucose control (AUC = 0.641) or with HbA1c < 6.5% (AUC = 0.668).

**Conclusions:**

TIR was independently associated with severity in patients with AP, particularly those with good antecedent glucose control.

## Introduction

Acute pancreatitis (AP), an inflammatory injury of pancreatic edema, bleeding, and necrosis, is the leading cause of hospital admission for gastrointestinal disorders in China and many other countries. According to the new Atlanta Classification of 2012 revision, AP can be classified into mild acute pancreatitis (MAP), moderately severe acute pancreatitis (MSAP), and severe acute pancreatitis(SAP) [1]. Approximately 20% of patients develop moderate or severe acute pancreatitis, with multiple organ dysfunction or local complications, and a substantial mortality rate of 20–40% [2]. As a progressive disease, AP has the risk of developing from mild to severe, even during the hospital stay [3]. Hence, it is essential to explore the factors related to prognosis in the early stages of severity to decrease serious complications and mortality rates.

A growing number of studies have focused on the association between blood glucose levels and AP outcomes of acute pancreatitis. A post-hoc analysis of a prospective, international cohort of 2250 acute pancreatitis cases demonstrated that both on-admission and peak serum glucose levels were independently associated with AP severity [4]. Another prospective study found that the blood glucose level on admission (> 11.1 mmol/L) was a significant risk factor for worsening of AP [5]. However, the blood glucose indicators used in these studies, including mean and maximum blood glucose, glycosylated hemoglobin (HbA1C), and blood glucose on admission, failed to accurately reflect glycemic fluctuation during the onset.

Poor glycemic control was considered as an independent risk factor for the deterioration in acute severe diseases [6, 7]. Studies pertaining to glycemic control in critically ill patients have recently focused on the target blood glucose to determine the optimal level of blood glucose.

The blood glucose percentage of time in range (TIR) is a new metric suggested as the unifying metric to account for hypoglycemia, glycemic variation, and hyperglycemia events [8]. Recent research has recognized the critical role played by TIR as a prognostic factor for critically ill patients [9–12], and the impact of TIR varies among patients with different antecedent glucose control. However, little is known about the association between TIR in early stage and the progression of acute pancreatitis.

Accordingly, we conducted a retrospective study to investigate the association between TIR at 72 h after admission as a surrogate marker of glycemic control in the early stage and disease progression in acute pancreatitis.

## Methods

The study was approved by the ethics committee of the Affiliated Changzhou No.2 People’s Hospital of Nanjing Medical University and was performed in patients initially diagnosed with acute pancreatitis, who were admitted to the gastroenterology department between January 2017 and December 2021. All the selected individuals fasted for the first 72 h. We excluded individuals younger than 18 years and those with diabetic ketoacidosis. Individuals with organ failure at admission, length of hospital stay of less than three days, number of blood glucose monitoring less than 4 times per 24 h in the first 72 h, or pregnant were also ruled out. Data on sex, body mass index (BMI), age, etiology, organ failure, and history of diabetes were collected. Maximum C-reactive protein (CRP_max_),triglyceride(TG), and HbA1c levels were measured.

The nurses used glucose monitors (GLUPAD, SINOMEDISITE, China) to test capillary blood every one to six hours. None of participants received intravenous nutrition in the first 72 h on admission.We started continuous intravenous (IV) insulin when two consecutive BG readings ≥ 180 mg/dl, and monitor blood sugar every hour to prevent a rapid blood glucose decent. IV concentrated dextrose when blood glucose levels are < 70 mg/dL. We collected all blood glucose values of the individuals in the first 72 h of admission. Using the measured blood glucose values, we calculated the mean blood glucose and TIR (70–180 mg/dL, which represented the percentage of time between 70 and 180 mg/dL) in the first 72 h. Clinically significant hypoglycemia was defined as a glucose level < 54 mg/dL. The coefficient of variation (%CV),which represents glycemic variability, resulted from the division between the standard deviation and average glucose multiplied by 100.

AP was diagnosed if two of the following three criteria were met clinically:(1) acute, sudden, continuous, and severe upper abdominal pain, which can radiate to the back; (2) serum amylase and/or lipase activities were at least three times higher than the upper limit of normal; (3) Enhanced CT / MRI showed typical imaging changes of AP (pancreatic edema or peripancreatic effusion).

Individuals were divided into two groups according to the severity of pancreatitis at discharge diagnosis:(1) mild group: patients who met the diagnostic criteria for AP without organ failure and local or systemic complications during hospitalization; (2) severe group, including MSAP and SAP, accompanied by transient organ failure (which can be recovered within 48 h), or accompanied by single or multiple organ failure continuously (> 48 h duration).

## Statistics

Continuous data are displayed as median (interquartile range (IQR)) or mean (standard deviation(SD)), and comparisons were made between groups using the Mann–Whitney rank-sum test or Student’s t-test, respectively, as appropriate. Categorical data are presented as percentages, and comparisons were made between groups using the χ^2^ test. TIR was further divided into categories based on quartiles (Q1 < 40.88%, Q2 40.89–70.18%, Q3 70.19–85.71, Q4 ≥ 85.72). Mantel–Haenszel χ^2^ test to investigate linear trend of the association between the prevalence of severe rates and TIR quartiles.Kruskal Wallis one way ANOVA was used to compare the CRP_max_ level in each group.

Logistic regression analysis was conducted to investigate the independent association between the TIR and severity. The Receiver Operating Characteristic Curve (ROC) was used to detect the predictive accuracy of TIR on severity.

## Results

**Fig. 1:**
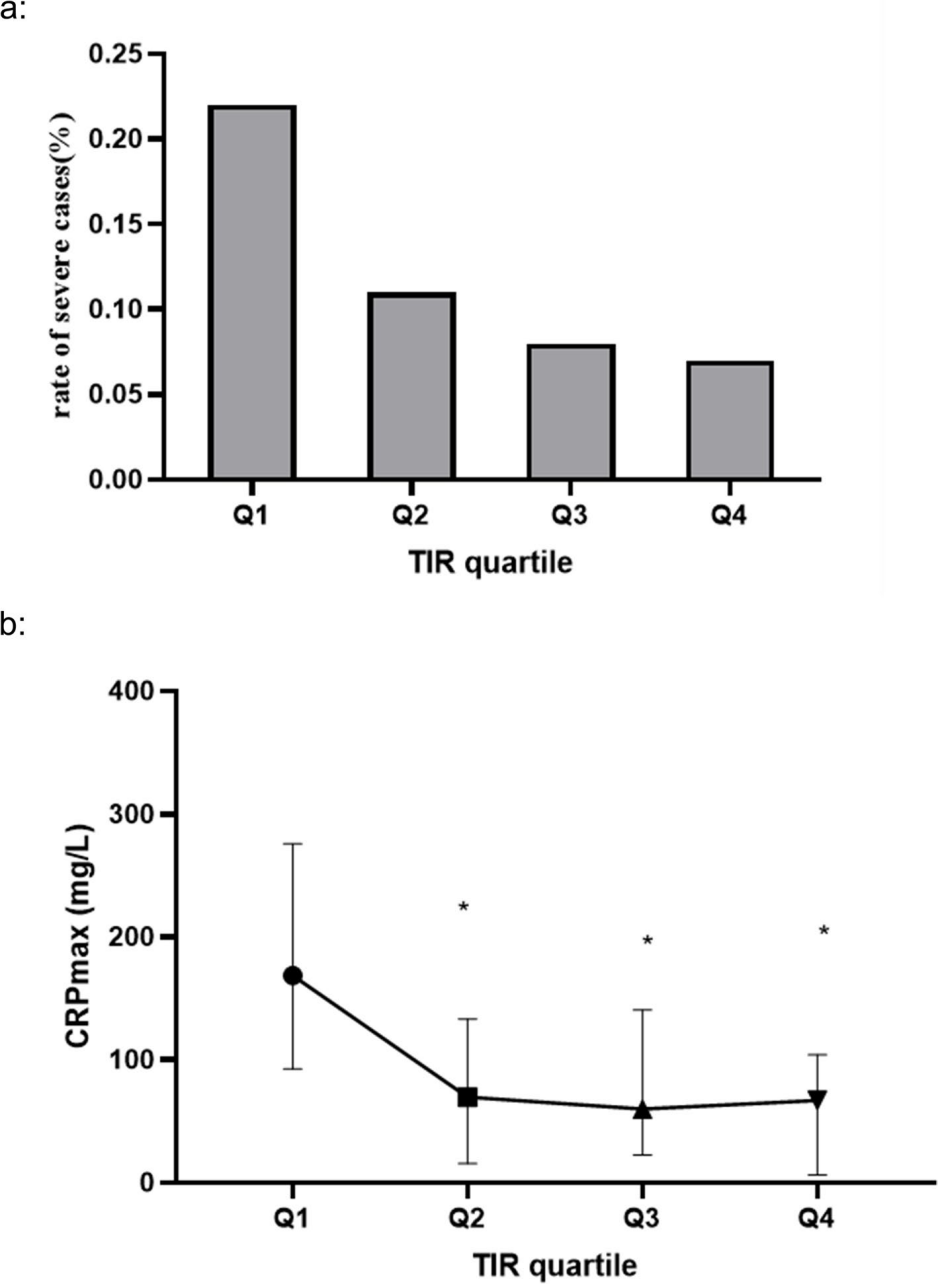
**a** Rate severe cases according to TIR quartile (p for trend < 0.05). **b** CRPmax level according to TIR quartile (*VS Q1 *p* < 0.05)

In total, 298 individuals were included in the analysis. 35 developed MSAP or SAP while in the hospital, of whom 24 developed MSAP and 11 developed SAP (Table 1). Compared with their non-severe counterparts, individuals in the severe group(MSAP or SAP) had a higher proportion of diabetes and extreme hypoglycemia, higher mean blood glucose values, HbA1c, and TG levels (all *p* < 0.05). The TIR of the severe group and the proportion of TIR > 70% were also lower (*p* < 0.05). When using TIR as a categorical variable based on the quartile of individuals (< 40.88%, 40.89%-70.18%, 70.19%-85.71%, and ≥ 85.72%), as illustrated in Fig. 1a, the prevalence of severe cases decreased progressively across the categories of increasing TIR (p for trend < 0.001). As shown in Fig. 1b, a trend of decreasing CRP_max_ levels was also observed (p for trend < 0.001). The CRP_max_ levels in the lowest quartile group of TIRwere significantly higher than those in the other groups. (*p* < 0.05).

**Table 1:** Characteristics of patients who developed more severe pancreatitis or not

	Total (*N* = 298)	MAP (*N* = 263)	SAP or MSAP (*N *= 35)	*p*
BMI (Kg/m^2^)	25.79 ± 4.39	25.73 ± 4.35	26.25 ± 4.77	0.540
Age (year)	48.67 ± 15.72	48.16 ± 15.14	52.49 ± 19.44	0.211
Sex (male%)	207 (69.46)	181 (68.82%)	26 (74.29%)	0.510
HbA1C (%)	7.12 ± 2.27	7.03 ± 2.22	7.81 ± 2.51	0.054
TG (mmol/L)	2.27 (0.99, 5.81)	2.22 (0.98–5.21)	4.07 (1.30–8.90)	0.041
CRP max (mg/L)	79.75 (25.93, 160.98)	74.30 (20.75, 146.9)	183.17 (88.03,309.99)	< 0.01
Diabetes n (%)	102 (34.22)	83 (31.56)	19 (54.29)	< 0.01
Heart failure n (%)	24 (8.05)	4 (1.52)	20 (57.14)	< 0.01
Kidney failure n (%)	30 (10.07)	15 (5.70)	15 (52.86)	< 0.01
Respiratory failure n (%)	15 (5.03)	1 (0.38)	14(40.00)	< 0.01
**Etiology**				
Biliary n (%)	129 (43.29)	114 (43.45)	15 (42.86)	
HTG n (%)	79 (26.51)	64 (24.33)	15 (42.86)	
Alcohol n (%)	37 (12.41)	33 (12.55)	4 (11.43)	0.029
Combined n (%)	53 (30.20)	52 (19.77)	1 (2.86)	
**Glucose control**				
Mean median (IQR)	8.76 (7.56,10.99)	8.61 (7.48,10.71)	10.35 (8.38,13.04)	< 0.01
Hypoglycemia n (%)	11 (3.69)	7 (2.66)	4 (11.43)	0.029
CV % median (IQR)	27.44 (21.37, 33.44)	27.69 (21.53, 33.67)	24.02(20.51, 30.55)	0.145
TIR median (IQR)	70.19 (40.88, 85.71)	71.43 (44.62, 86.67)	55.41 (12.82, 77.78)	< 0.01
TIR ≥ 70% n (%)	112 (37.58)	105 (39.93)	7 (20.00)	0.022
CV > 36% n (%)	53 (17.79)	47 (17.87)	6 (17.14)	0.600

*MAP* mild pancreatitis, *MSAP* moderately severe acute pancreatitis, *SAP* severe acute pancreatitis, *TIR* time in range, *BMI* body mass index, *TG* triglyceride, *HbA1C* hemoglobin A1C, *HTG* Hypertriglyceridemia, *CV* coefficient of variation, *CRP C*-reaction protein, *IQR* interquartile range

Logistic regression analysis revealed that TIR was significantly associated with the risk of developing,SAP or MSAP in the crude and adjusted models, including age, sex, BMI,TG, HbA1c, diabetes, and etiology as covariates (OR = 0.962, 95% CI = 0.941–0.983, *p* = 0.001), as shown in Table 2. This association remained significant with severity in individuals with HbA1c < 6.5% after adjusting for age, sex, BMI,TG, diabetes, and etiology (OR = 0.928, 95% CI = 0.888–0.969, *p* = 0.001). In contrast, in individuals with HbA1c ≥ 6.5%, there was no significant association between TIR and severity in either the unadjusted or adjusted logistic regression model (OR = 0.971,95% CI = 0.942–1.000, *p* = 0.053).

**Table 2:** Association of TIR 70-180 mg/dl in first 72 h with MAP or MSAP by logistic regression analyses

	**Models**	**OR**	**95%CI**	***p***
**ALL**	crude	0.981	0.969–0.993	0.001
	Model 1	0.968	0.953–0.983	< 0.001
	Model 2	0.962	0.941–0.983	0.001
**HbA1C < 6.5%**	crude	0.952	0.925–0.981	0.001
	Model 1	0.936	0.905–0.969	< 0.001
	Model 2 ^a^	0.928	0.888–0.969	0.001
**HbA1C ≥ 6.5%**	Crude	0.986	0.964–1.008	0.212
	Model 1	0.967	0.941–0.995	0.019
	Model 2 ^a^	0.971	0.942–1.000	0.053

Model 1: is adjusted for age, sex and body mass index

Model 2: includes all variables in Model 1 plus HAb1C, TG, Diabetes and etiology of AP

Model 2 ^a^: includes all variables in Model 1 plus TG, Diabetes and etiology of AP

In Table 3, a TIR of 70–180 mg/dl ≥ 70% in the first 72 h was independently associated with reduced severe cases only in individuals with well-antecedent controlled blood glucose after adjusting for age, sex, BMI,TG, diabetes, and etiology(OR = 0.238; 95% CI = 0.071–0.802; *p* = 0.020).

**Table 3:** Association of TIR 70-180 mg/dl ≥ 70% in first 72 h with MAP or MSAP by logistic regression analyses

	**Models**	**OR**	**95%CI**	***p***
**ALL**	crude	0.409	0.192–0.869	0.020
	Model 1	0.289	0.122–0.684	0.005
	Model 2	0.418	0.146–1.195	0.103
**HbA1C < 6.5%**	Crude	0.319	0.115–0.886	0.028
	Model 1	0.231	0.077–0.699	0.010
	Model 2 ^a^	0.238	0.071–0.802	0.020
**HbA1C ≥ 6.5%**	Crude	0.917	0.187–4.490	0.915
	Model 1	0.605	0.103–3.568	0.579
	Model 2 ^a^	1.207	0.157–9.280	0.856

Model 1: is adjusted for age, sex and body mass index

Model 2: includes all variables in Model 1 plus HAb1C, TG and etiology of AP

Model 2 ^a^: includes all variables in Model 1 plus TG, Diabetes and etiology of AP

**Fig. 2:**
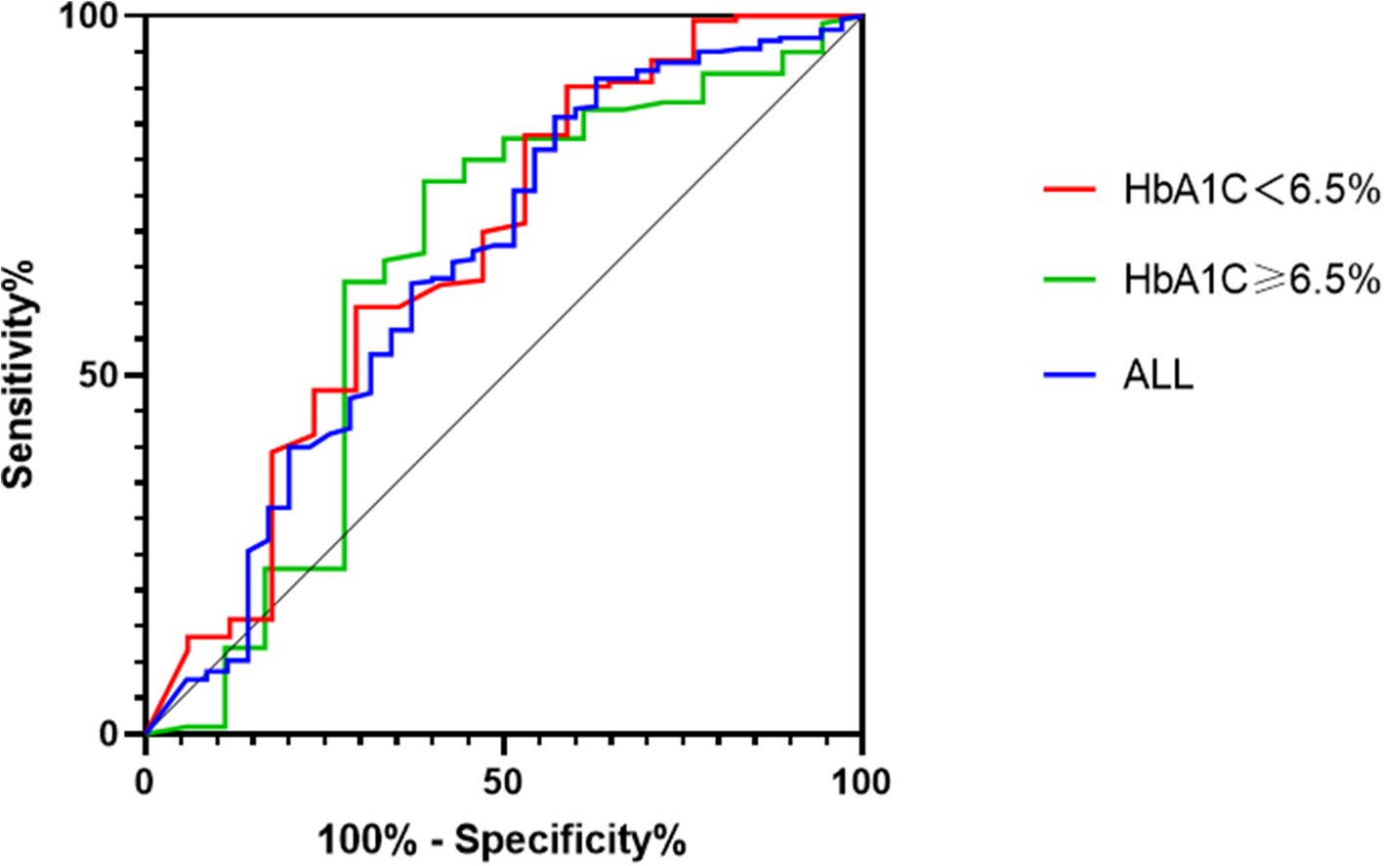
The predictive ability of TIR for severe pancreatitis in people with different blood glucose status

We assessed prediction probabilities by comparing the area under the receiver operating characteristic curve (AUC). The ROC analysis (Fig. 2) showed that TIR as a continuous variable failed to predict severity in individuals with HbA1c < 6.5%(AUC = 0.668) or ≥ 6.5% (AUC = 0.641). It was not a powerful predictor of overall severity, regardless of antecedent blood glucose (AUC = 0.647).

## Discussion

This single-center retrospective analysis of patients with AP revealed that blood glucose in the TIR 70–180 mg/dL in the first 72 h on admission is strongly associated with a decreased incidence of severe cases in well-controlled individuals. This association was not observed in their poorly controlled counterparts. according to the logistic regression analysis.

These data expand the limited literature that has explored the relationship between glucose control and AP progression. Several reports have shown that patients with DM have a greater incidence of acute pancreatitis compared than non-diabetics [13, 14], indicating that long-term poorly controlled blood glucose may lead to the occurrence of pancreatitis. In mouse models, it was observed that diabetes aggravated acute pancreatitis and suppressed regeneration of exocrine tissue [15]. Possible mechanisms include hyperlipidemia [16], a pre-inflammatory state [15], immune dysfunction, and oxidative stress caused by chronic hyperglycemia. On the other hand, pancreatitis could cause fluctuations in blood glucose, and mechanisms may lie in: first, stress hyperglycemia that mainly occurs in the early stage of onset, and a variety of inflammatory factors stimulate the increase in the release of glucagon, glucocorticoid, and other hyperglycemic hormones; second, microcirculation disturbance of pancreatic tissue characterized by edema, ischemia, and necrosis that result in destruction of islets, after which a large amount of insulin release and insufficient nutrition can lead to hypoglycemia. However, the association between glycemic levels in the early stages and the outcome of pancreatitis has rarely been reported. Nagy et al. [4] found that, on admission, peak in-hospital serum glucose concentrations had a significant dose-dependent relationship with AP severity after adjusting for DM, age, sex, and etiology. Additionally, a hospital glucose peak > 7 mmol/L was associated with a 15 times higher probability of severe AP. Another retrospective study [17] showed that the glycemic lability index upon admission contributed independently to the risk of mortality,which was better able to predict the death of patients with severe pancreatitis than the mean glucose level.

As a suitable metric for the efficacy and safety of glycemic control, TIR has recently received increasing attention, with many studies suggesting that it has an important correlation with the prognosis of severe disease. In previous studies exploring the relationship between TIR and mortality in critically ill patients, Matthew Signal discovered that a TIR of 72–126 mg/dl ≥ 50% was independently associated with reduced organ failure [18]. They also found that TIR ≥ 70% was independently associated with increased chances of survival over TIR ≥ 50% or TIR ≥ 30% in a further study [19]. We observed that in the first 72 h of admission, patients who developed SAP or MSAP had significantly lower levels of TIR, as well as a lower proportion of patients with TIR above 70%. In other words, patients with a TIR greater than 70% at an early stage are less likely to progress to SAP or MSAP. CRP is considered as one of the biomarkers reflecting the severity of the inflammation [20]. Given that TIR has a statistically significant dose-dependent relationship with maximal CRP level, we supposed that patients with extremely low TIR at an early stage may suffer from more severe inflammation.

The patients were further divided into two groups according to antecedent glucose control, as reflected by HbA1c level. In patients with HAb1c less than 6.5%, TIR in the first 72 h was negatively associated with the occurrence of SAP or MAP, regardless of the adjusted confounders. No apparent association was found in patients with HbA1c levels of ≥ 6.5%. Similarly, well-controlled patients with TIR 70–180 mg/dL greater than 70% in the first 72 h were less likely to progress to SAP or MSAP, while patients with HbA1c ≥ 6.5% under the same conditions did not benefit.

The observed association of TIR in the early stage and outcome of severity in patients with AP with varied antecedent glucose control was similar to those of earlier studies on critically ill patients and mortality. Krinsley et al. [10] demonstrated that TIR 70–140 mg/dl > 80% is strongly associated with survival in critically ill patients without diabetes, while no difference was found in mortality between groups among diabetic patients. Lanspa et al. [21] found that a TIR of 70–139 mg/dl > 80% was independently associated with mortality in critically ill patients, particularly those with HbA1c < 6.5%, which represented well-controlled glucose levels before admission. Naraba et al. [9] chose a less stringent 70–180 mg/dl as the time in range,which was the same range we selected according to the guidelines from the Society of Critical Care Medicine and American Diabetes Association [22], and reported that lower TIR was associated with higher 28-day mortality in critically ill patients with HbA1c < 6.5%, whereas there was no consistent association in patients with HbA1c ≥ 6.5%. Furthermore, a similar association was found during the first three days in their study. Despite the slight difference in the chosen target range, our research further supports the idea that tight glycemic control might benefit patients without a history of diabetes or with well-controlled glucose levels in patients with AP.

One possible explanation is hyperglycemia adaption [23]. Chronic hyperglycemia can lead to the production of reactive oxygen species (ROS) [24], which may also play a key role in the progression of pancreatic inflammation. Patients with poor long-term blood glucose control have adapted to the adverse consequences induced by chronic hyperglycemia. However, patients with well-controlled blood glucose levels have poor tolerance to oxidative stress-related tissue injury induced by acute hyperglycemia and inflammation during AP, resulting in a more severe condition. Patients with DM can also better withstand relatively low glucose values because of the mechanism of cellular adaptation to recurrent hypoglycemia [25, 26]. As the incidence of in-hospital hypoglycemia in our study was extremely low, the effects beyond the target range were mainly due to hyperglycemia. These results remind us that antecedent control of AP should be taken into consideration when setting blood glucose targets. Further intervention studies are warranted for the appropriate management of acute pancreatitis patients with different blood glucose levels.

Regardless of whether TIR at an early stage was independently associated with the occurrence of SAP or MSAP,it was not a powerful predictor of such an outcome, as the area under the ROC curve of TIR was not large enough in all groups(from 0.641 to 0.668). In other words, the extent of deterioration of AP prognosis should not be entirely attributed to blood glucose levels. Indeed, it seems impossible for a biochemical variable to predict the outcome of AP.

To our knowledge, this is the first study to demonstrate the effect of TIR in the early stages of AP on the outcome. There were also some limitations to this study. First, because of the retrospective nature of the study, we could not formulate plans for the timing and frequency of the blood glucose measurements in advance. Second, owing to the characteristics of a single-center observational study, the conclusion may not be generalizable to all patients with AP. Third, the TIR in this study was not generated by a continuous glucose monitoring system (CGMS), which may reduce the accuracy, although we selected patients with relatively high-frequency measurements as far as possible. Hence, prospective studies with larger sample sizes are warranted to provide further evidence for optimal blood glucose control.

We demonstrated that a higher TIR of 70–180 mg/dL in the early stages of AP was associated with a lower risk of developing SAP or MSAP. Patients who maintained blood glucose levels between 70 and 180 mg/dL at least 70% of the time had a decreased possibility of developing SAP or MSAP compared to those who did not. All these findings were also found in individuals with good antecedent control, but not in poor antecedent controlled individuals, suggesting that patients with previously well-controlled blood glucose might benefit from early tight blood glucose management. Further interventional trials are needed to investigate the optimal blood glucose control target in patients with pancreatitis.

## Data Availability

The data generated during and/or analysed during the current study are available from the corresponding author on reasonable request.
